# A bispecific monomeric nanobody induces spike trimer dimers and neutralizes SARS-CoV-2 in vivo

**DOI:** 10.1038/s41467-021-27610-z

**Published:** 2022-01-10

**Authors:** Leo Hanke, Hrishikesh Das, Daniel J. Sheward, Laura Perez Vidakovics, Egon Urgard, Ainhoa Moliner-Morro, Changil Kim, Vivien Karl, Alec Pankow, Natalie L. Smith, Bartlomiej Porebski, Oscar Fernandez-Capetillo, Erdinc Sezgin, Gabriel K. Pedersen, Jonathan M. Coquet, B. Martin Hällberg, Ben Murrell, Gerald M. McInerney

**Affiliations:** 1 Department of Microbiology, Tumor and Cell Biology, Karolinska Institutet, Stockholm, Sweden; 2 Department of Cell and Molecular Biology, Karolinska Institutet, Stockholm, Sweden; 3 Division of Medical Virology, Institute of Infectious Diseases and Molecular Medicine, University of Cape Town, Cape Town, South Africa; 4 Science for Life Laboratory, Division of Genome Biology, Department of Medical Biochemistry and Biophysics, Karolinska Institutet, Stockholm, Sweden; 5 Genomic Instability Group, Spanish National Cancer Research Centre (CNIO), Madrid, 28029 Spain; 6 Science for Life Laboratory, Department of Women’s and Children’s Health, Karolinska Institutet, Stockholm, Sweden; 7 Center for Vaccine Research, Statens Serum Institut, Copenhagen, Denmark; 8 Karolinska Institutet VR-RÅC, Centre for Structural Systems Biology, Notkestraße 85, 22607 Hamburg, Germany

**Keywords:** Viral infection, SARS-CoV-2

## Abstract

Antibodies binding to the severe acute respiratory syndrome coronavirus 2 (SARS-CoV-2) spike have therapeutic promise, but emerging variants show the potential for virus escape. This emphasizes the need for therapeutic molecules with distinct and novel neutralization mechanisms. Here we describe the isolation of a nanobody that interacts simultaneously with two RBDs from different spike trimers of SARS-CoV-2, rapidly inducing the formation of spike trimer–dimers leading to the loss of their ability to attach to the host cell receptor, ACE2. We show that this nanobody potently neutralizes SARS-CoV-2, including the beta and delta variants, and cross-neutralizes SARS-CoV. Furthermore, we demonstrate the therapeutic potential of the nanobody against SARS-CoV-2 and the beta variant in a human ACE2 transgenic mouse model. This naturally elicited bispecific monomeric nanobody establishes an uncommon strategy for potent inactivation of viral antigens and represents a promising antiviral against emerging SARS-CoV-2 variants.

## Abstract (web summary)

Here, the authors isolate and characterize a bispecific monomeric nanobody that induces dimerization of SARS-CoV-2 spike trimers, neutralizes variants of concerns as well as SARS-CoV, and inhibits SARS-CoV-2 infection in mice.

## Introduction

The SARS-CoV-2 pandemic and the vast societal and economic consequences of the connected lockdowns have had devastating consequences for the world’s populations. Despite rapid progress in vaccine development, some emerging virus variants appear to be less effectively neutralized by antibodies elicited by the first-generation vaccines^1^ and the emergence of further variants is expected. To prepare for different scenarios and to have emergency alternatives at hand, novel ways to inhibit and neutralize the virus and Variants of Concern (VoC) are needed. As therapeutic agents in the early phases of SARS-CoV-2 infection, camelid-derived single-domain antibody fragments are promising candidates. These so-called ‘nanobodies’ not only bind their antigen with very high specificity and affinity, but they can also be easily produced to large quantities in *E. coli*^2^ and show favorable biochemical characteristics, including good thermal stability. Importantly, they can easily be combined to form homo- and heterodimers or other multimers to increase potency and target multiple epitopes simultaneously^3–5^. This strategy allows the neutralization of multiple known or unknown virus variants and reduces the risk for viral escape^5^.

The attachment of SARS-CoV-2 to the host cell receptor ACE2 and subsequent membrane fusion is mediated by the viral spike (S) protein^6^. In the prefusion conformation, the spike is a large homotrimer. Each S monomer consists of two subunits: S1 and S2. The S1 subunit contains the receptor binding domain (RBD) and S2 contains the fusion peptide, as well as the transmembrane domain. The RBD is responsible for the initial attachment to ACE2 and is found in two distinct conformational states: The receptor-accessible ‘up’ conformation and the receptor inaccessible ‘down’ conformation^6,7^. Receptor binding induces the rather unstable 3-up state, which leads to shedding of S1 and refolding of S2 thereby presenting the fusion peptide to the opposing membrane^6,8,9^. SARS-CoV-2 is easily neutralized by high-affinity binding antibodies and other spike-targeting molecules, including nanobodies^10–13^. Many of them bind to, or close to, the receptor binding motif of the RBD and neutralize by directly hindering virus attachment^14,15^. Other mechanisms include locking the RBDs in the down conformation or preventing productive fusion^5,16^. Still, the emergence of new virus variants that evade antibody-mediated neutralization mandates the identification of molecules with a more diverse set of binding epitopes and neutralization mechanisms allowing for neutralization of a variety of virus variants. Here, we generated a nanobody that has two nonoverlapping interaction sites on the SARS-CoV-2 RBD, each targeting a different epitope, which induces the formation of dimers of spike trimers and rescues mice from lethal SARS-CoV-2 infection.

## Results

### Isolation and characterization of a potent RBD-specific nanobody

**Fig. 1:**
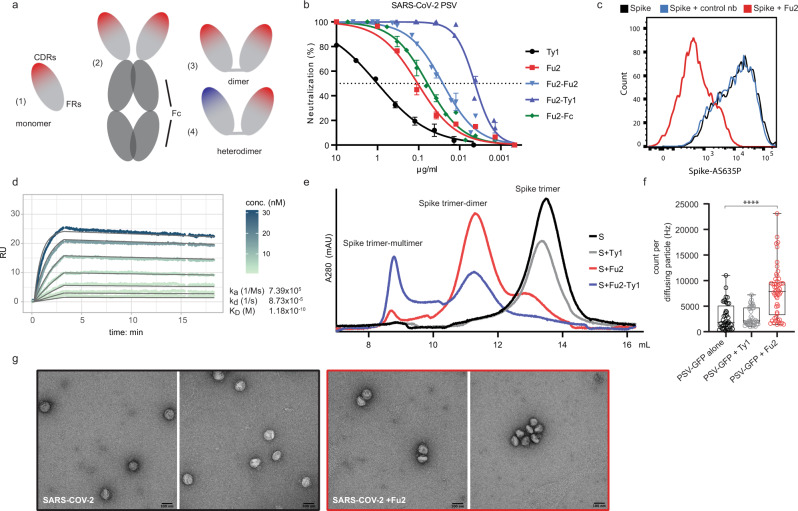
An RBD-specific nanobody neutralizes SARS-CoV-2. **a** Overview of different nanobody constructs used in this study: (1) nanobody monomer, (2) nanobody-Fc fusion, (3) chemically linked nanobody homodimer, (4) chemically linked nanobody heterodimer. **b** A SARS-CoV-2 spike pseudotyped lentivirus (PSV) was incubated with a dilution series of the indicated nanobodies at 37 °C for 1 hour before infecting HEK293T-hACE2 cells. Neutralization (in %) compared to untreated PSV is shown. Data from two (Fu2) or three (all others) replicates is shown. Data are presented as mean values and standard deviation. Fu2-Fu2 and Fu2-Ty1 refer to the homodimeric and heterodimeric constructs respectively, if just Fu2 or Ty1 is indicated, the monomeric nanobody was used. b with the *x*-axis in nM is shown in Fig. S1. **c** Recombinantly expressed, prefusion stabilized, and fluorescently labelled SARS-CoV-2 spike protein was incubated with a control nanobody specific for IAV NP, or Fu2 and used to stain ACE2 expressing HEK293T cells. Cells were analyzed by flow cytometry and a representative histogram is shown. **d** Binding kinetics of Fu2 to the RBD were measured by surface plasmon resonance (SPR). Sensorgram is color-coded based on concentration. The fit is based on the 1:1 Langmuir model and is shown in dark grey solid lines. **e** Recombinantly expressed, prefusion-stabilized SARS-CoV-2 spike protein was run alone or preincubated with the indicated nanobody constructs on a Superose 6 size-exclusion column. Elution profiles (A280) are shown. **f** GFP-labelled SARS-CoV-2 spike pseudotyped lentivirus (PSV-GFP) was incubated with Ty1, Fu2 or left alone and brightness of diffusing virus particles was quantified using fluorescence correlation spectroscopy in solution. Box-and-whisker plots show all data points (as circles) with median as the box center, lower and upper quartiles as the box boundaries and minimum (lower extreme) to maximum (upper extreme) points as error bars. Statistical significance was evaluated using two-sided Kolmogorov–Smirnov test. **g** SARS-CoV-2 was incubated with or without Fu2 and visualized by negative-stain electron microscopy. This experiment was performed once, and multiple images were taken from each condition. Source data are provided as a Source Data file for **b, d** and **e** and **f**.

To identify nanobodies with unique mode of neutralization, we immunized one alpaca (*Funny*) with recombinant SARS-CoV-2 spike protein and RBD. We isolated RNA from peripheral blood mononuclear cells and created phagemid libraries. Phage display screens with immobilized spike protein identified one promising nanobody, called Fu2. Since multimerization of nanobodies can substantially improve virus neutralization potency^3,17^, we created three different dimers (illustrated in Fig. 1a): An Fu2-Fc fusion expressed in mammalian cells, a chemically linked Fu2 homodimer, and thirdly, a heterodimer of Fu2 with a previously described RBD-specific neutralizing nanobody Ty1^14^. Chemically linked constructs were generated using a combination of sortase A labelling and Cu-free click-chemistry as described in detail previously^3^.

Using a pseudotyped lentivirus (PSV) neutralization assay (Fig. 1b), we found that Fu2 was ~10 times more potent than Ty1, with an IC_50_ in the range of 106 ng/ml (7 nM). The Fu2-Fc fusion only slightly increased the neutralization potency of this nanobody to 61 ng/ml (0.75 nM), and a chemical homodimerization to an IC_50_ of 26 ng/ml (0.8 nM). Thus, the observed enhancement in potency is attributable to dimerization, rather than the Fc-domain itself. Interestingly, the Fu2-Ty1 heterodimer was extremely potent, with an IC_50_ of 4 ng/ml (140 pM), which is approximately six-fold lower than that of the Fu2 homodimer. Fu2 directly prevented spike binding to ACE2 as confirmed by flow cytometry of HEK293T-hACE2 cells stained with recombinant, fluorescently labelled spike (±Fu2) (Fig. 1c). Analysis of binding kinetics by surface plasmon resonance (SPR) revealed that Fu2 bound the RBD with subnanomolar affinity (Fig. 1d). A 1:1 binding model fit the sensorgrams well at the used concentration range, indicating a single dominant interaction. Analysis of next-generation sequencing data for other members of the Fu2 lineage yielded several Fu2 variants with similar properties (Fig. S2a–f).

To test the integrity of the recombinant spike protein after the addition of Fu2, we analyzed the size-exclusion chromatography elution profiles in combination with different nanobody constructs (Fig. 1e). The SARS-CoV-2 spike trimer eluted in a single peak and the addition of Ty1^14^ did not result in a shift in the position of this peak, which is expected given the small relative size addition of the 14 kDa nanobody to the large (>400 kDa) spike trimer. In contrast, the addition of Fu2 resulted in a substantial shift of the peak, suggesting the formation of a larger complex, likely a dimer of the spike trimer. Next, we combined the spike with the Fu2-Ty1 heterodimer. This also resulted in earlier elution, and besides a possible spike dimer, this construct gave rise to an even earlier elution peak suggesting the induction of higher-order spike multimers.

To test if the Fu2-induced multimerization of spike also occurs when it is presented on virus particles, we analyzed GFP-labeled SARS-CoV-2 pseudotyped lentiviruses (PSV-GFP) after addition of the nanobodies Ty1 or Fu2 (Fig. 1f). To this end, we performed fluorescence correlation spectroscopy and measured apparent brightness of each diffusing particle. Most single diffusing particles preincubated with Fu2 showed a 2.5-fold increase in brightness compared to PSV-GFP alone or preincubated with Ty1, suggesting that Fu2 leads to aggregation of the PSV-GFP particles. Furthermore, negative-stain electron microscopy showed an increased number of dimerized or oligomerized SARS-CoV-2 virions in an Fu2-treated sample compared to a nontreated control (Fig. 1g).

### Structural basis for the induction of spike trimer–dimers by Fu2

**Fig. 2:**
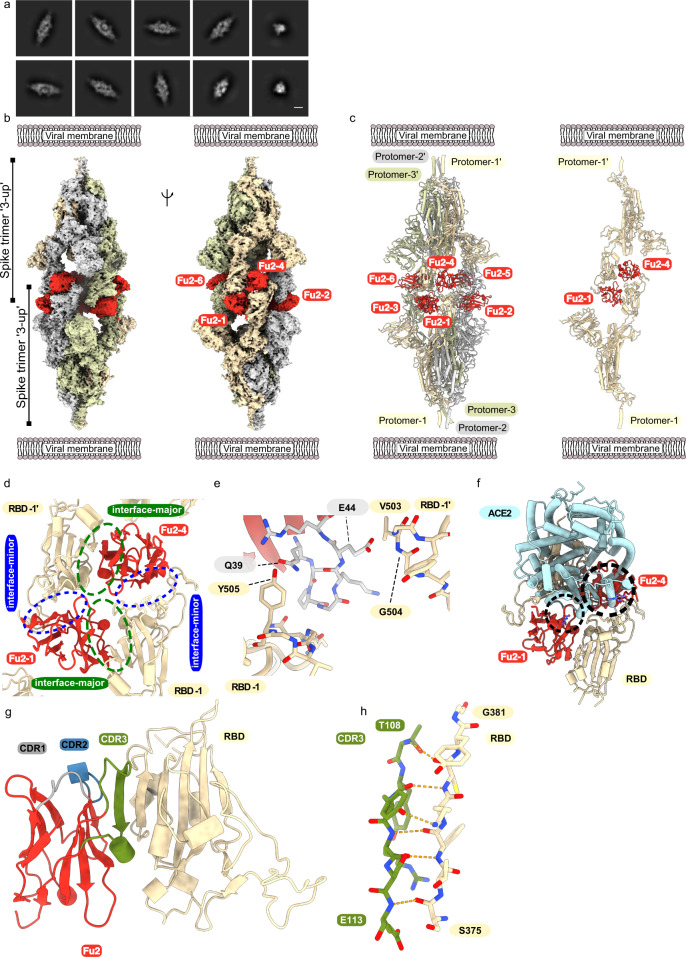
Cryo-EM provides structural evidence of Fu2-mediated dimerization of trimeric spike in ‘3-up’ conformation. **a** Representative 2D class averages (scalebar = 100 Å). **b** Cryo-EM map (two different side views), and **c** Atomic model of ‘dimeric’ spike in complex with Fu2; and two opposite protomers with two Fu2 molecules. Both spike trimers in the complex are present in ‘3-up’ conformation (six Fu2 molecules are numbered). **d** Closeup view of the RBD-Fu2 interaction interfaces showing the interface-major and interface-minor. **e** A β-hairpin (gray sticks) from the Fu2 framework region (residues 39–45) contacts two RBDs simultaneously in the Fu2-mediated spike trimer–dimer. **f** Modelling of simultaneous spike-Fu2-ACE2 binding shows that Fu2 blocks the binding of ACE2 (PDB:6LZG^18^) (https://www.rcsb.org/structure/6LZG) to the RBD from two directions. Clashes marked with dashed circles. **g** Fu2 binds the RBD through an extended interchain β-sheet interaction mediated by the CDR3 region (green). CDR1 (gray) and CDR2 (blue) does not take part in RBD binding. **h** Closeup of a part of the CDR3-RBD interface displaying the antiparallel β-strand hydrogen bond pairing between the Fu2 CDR3 region and the RBD (light orange).

To better understand the molecular basis for neutralization and aggregation of spike by Fu2, we used electron cryomicroscopy (cryo-EM) to determine the structure of the Fu2-spike complex. The structure revealed a remarkable head-to-head dimerization of the trimeric spike, bound by six molecules of Fu2 (Fig. 2). This distinct structural state appeared as prominent 2D classes in our analyses (Fig. 2a). Binding of Fu2 to the RBD is unique as it can simultaneously interact with two different RBDs from different spike trimers in the up conformation (Fig. 2b, c). Our high resolution (2.9 Å; 0.143 FSC) localized reconstruction of the RBD-Fu2-RBD-Fu2 interface helped us elucidate the molecular details of this interaction. Each RBD in the complex provides two binding interfaces for two Fu2 molecules, which we call ‘interface-major’ and ‘interface-minor’ according to surface area covered (Figs. 2d, S3a and S3b). The interface-major and interface-minor have a buried surface area of ~740 and ~495 Å^2^, respectively. Parts of the Fu2 framework regions drive the dimerization by shape-complementarity and coupled solvent exclusion at the two interfaces. In addition, the interface-major, which is likely responsible for the single dominant interaction determined by SPR, consists of 15 hydrogen bonds and one salt bridge between Fu2 D117 and RBD K378, while the interface-minor comprises five hydrogen bonds. Furthermore, a region around the Fu2 β-turn A40-K43 plays a key role in dimerization through interactions with two proximal RBDs (Figs. S3c, d). Specifically, one of the two proximal RBDs’ T500-Y505 region packs against the Fu2 A40-K43 β-turn and the RBD Y505 is in addition bound by Fu2 Q39. Meanwhile, the other proximal RBD is bound through a bidentate hydrogen bond between Fu2 E44 and the backbone amides of RBD V503-G504 (Figs. 2e, S3c, d). Interestingly, the overall structure of Fu2-bound spike had no significant deviations from previous structures (Fig. S4), and we did not find any contact between any two Fu2 molecules or between spike trimers suggesting that the observed spike trimer dimerization is solely driven RBD-Fu2 interactions.

### Fu2 blocks the RBD from binding ACE2

Structural alignment of an ACE2-RBD structure (PDB: 6LZG^18^) (https://www.rcsb.org/structure/6LZG) to the Fu2-spike structure (single RBD) shows that binding of ACE2 would be hindered by Fu2 bound to any of the two interfaces (Fig. 2f). Fu2 bound to the interface-major would clash with ACE2 residues ~303–331 and the N-glycan at position 322, while Fu2 bound to interface-minor would mask the interaction surface of ACE2, making it sterically impossible for ACE2 to access the RBD, suggesting that spike dimerization and virion aggregation is an additional mechanism and not strictly required for neutralization. A comparison of the Fu2 and Ty1 binding site revealed that interface-minor is partially shared between Ty1 and Fu2 (Fig. S3e, f), suggesting that in context of the Fu2-Ty1 heterodimer, Ty1 is displaced upon dimerization of the spike trimer. Ty1 can then potentially bind additional spike molecules, explaining the elution profile showing both dimers and higher-order spike complexes (Fig. 1e).

In the complex, Fu2’s long CDR3 (G99–Y120) plays a significant role in RBD binding. Here, the Fu2 S107-R111 region adopts a β-strand conformation that binds the RBD T376-Y380 β-strand to give rise to an energetically favorable interchain extension of the RBD antiparallel β-sheet (Fig. 2g, h). Interestingly, the Fu2 CDR1 (G26–Y32) and CDR2 (T52–S57) do not participate in RBD binding, and none of the CDRs is directly involved in the formation of the spike dimer.

### Fu2 stabilizes the RBD in the ‘up’ conformation

Cryo-EM analyses, both of purified spike or of intact virions, have revealed that unliganded spike is mostly present in all-down (~60%) or 1-up (~40%) conformation66. Our Fu2-spike structure shows the presence of two trimeric spike molecules, both in the 3-up conformation. This arrangement allows Fu2 to access all six possible binding sites on RBDs without steric conflict. Alignment of RBD-Fu2 to RBD-down to a 2-up spike structure confirms that binding of Fu2 to RBD in the down conformation is not possible (Fig. S3g). Fu2 bound to either interaction interface on an RBD in the down conformation would clash with neighboring RBDs. Likewise, an alignment of the 2-up spike structure to the Fu2-trimer-dimer shows that RBD-down would clash with the neighboring Fu2 molecule (Fig. S3h). In conclusion, for Fu2 binding and for trimer–dimer assembly, all RBDs are bound in the up conformation and consequently then locked in this conformation.

### Fu2 does not target unique epitopes on the RBD

To understand how Fu2 interacts with the RBD compared to other published RBD-specific antibodies and nanobodies, we analyzed and compared their binding sites (Fig. S5). The S309 Fab fragment^19^ can bind to SARS-CoV-2 spike RBD in both up and down conformation and does not share the binding surface with Fu2. Likewise, monoclonal antibodies 47D11^20^ and S2M11^21^ bind on a distinct surface on RBD and do not share the binding surfaces with Fu2. In contrast, the monoclonal antibody CR3022^22,23^ partially overlaps with the interface-major, while antibody C144^24^ binding overlaps with interface-minor. Nanobody Wnb2 and Wnb10^25^ bind to the RBD in the up conformation and partially share the interface-major and interface-minor of Fu2, respectively. Similarly, nanobody VHH72^26^ partially shares the interface-major with Fu2. In conclusion, other antibodies and nanobodies have been described to bind to similar epitopes as Fu2 and targeting this binding site alone does not appear to responsible for induction of spike trimer–dimers.

### Fu2 neutralizes variants of concern and cross-neutralizes SARS-CoV

**Fig. 3:**
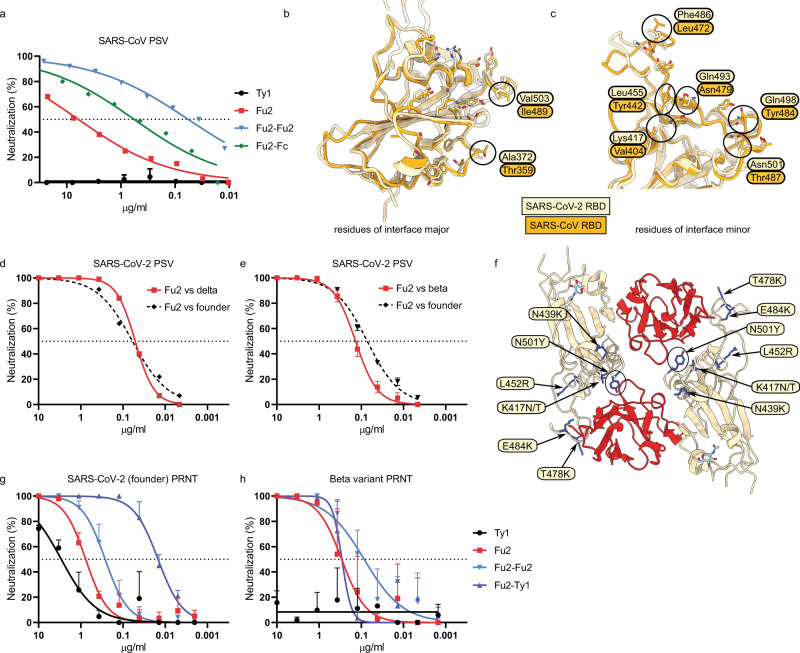
Fu2 cross-neutralizes SARS-CoV and SARS-CoV-2 variants of concern. **a** SARS-CoV S pseudotyped lentivirus (PSV) was incubated with the indicated constructs before infecting HEK293T-ACE2 cells, and neutralization is shown compared to untreated PSV. Error bars indicated means values + SD. **b**, **c** Structural basis of SARS-CoV cross-neutralization by Fu2. Structural alignment of SARS-CoV-2 Spike (RBD bound Fu2) with SARS-CoV RBD (PDB:6ACD). Residues of interface-major and interface-minor shown as sticks, divergent residues are labelled. **d**, **e** Comparison of Fu2 neutralization of founder PSV with delta (**d**) or beta (**e**) PSV. Error bars indicated means values + SD. **f** Assessment of Fu2-RBD interaction in RBD variants. In silico mutational analyses of RBD residues and probable impact on Fu2 binding. Fu2 is shown in red and variant residues are indicated. **g, h** PRNTs with clinical isolates of SARS-CoV-2. 100 plaque-forming units (PFU) of SARS-CoV-2^27^ or the beta variant^1^ were incubated with dilution series of the indicated nanobody constructs for 1 h at 37 °C before infecting monolayers of Vero E6 cells. Plaques were quantified 72 h post infection. Neutralization representing the reduction of plaques relative to the control wells is shown. Error bars represent mean + SD across three replicate experiments. Source data are provided as a Source Data file for **a, d, e, g**, and **h**.

To test the breadth of neutralization, we evaluated cross-neutralization of SARS-CoV PSV (Fig. 3a). While Ty1 did not neutralize SARS-CoV, Fu2 and dimeric Fu2 constructs were neutralizing, but less so than against SARS-CoV-2 PSV. Interestingly, the neutralization curves displayed a much flatter slope as compared to those against SARS-CoV-2 PSV. It required 6.1 μg /ml of monomeric Fu2 to reach 50% neutralization in this assay, while 570 ng /ml of Fu2-Fc and 57 ng/ml of the Fu2 dimer was sufficient for 50% neutralization.

To understand the molecular basis for cross-neutralization of SARS-CoV we compared the RBD sequences and performed structural alignments (Figs. 3b, c and S5a). Interestingly, the interface-major residues are conserved, while interface-minor has significant differences, suggesting that the divergent interface-minor of SARS-CoV RBD would not permit Fu2 binding. From these analyses we conclude that Fu2 would be unlikely to induce SARS-CoV trimer–dimers.

SARS-CoV-2 variants are rising in frequency and confer escape from many existing monoclonal antibodies and nanobodies. We evaluated the neutralization potential of Fu2 against two VoCs using PSV (Fig. 3d, e). Lentiviruses pseudotyped with the spike of either the beta or the delta variant were neutralized by Fu2 similarly as lentivirus pseudotyped with the spike of the founder virus, suggesting no significant reduction in neutralization potential against these variants.

To demonstrate neutralization of replication-competent SARS-CoV-2 we performed a plaque reduction neutralization test (PRNT) using infectious SARS-CoV-2, isolated from the first Swedish COVID-19 patient^27^ (Fig. 3g). Similar to neutralization of PSV, monomeric Fu2 neutralized SARS-CoV-2 much better than Ty1, a dimeric construct of Fu2 provided a 2.8-fold improvement over the monomeric version of Fu2, while the heterodimer of Ty1 and Fu2 showed a 50-fold improvement compared to monomeric Fu2 in this assay.

A PRNT using an isolate of the beta variant^1^ showed that Fu2 very potently neutralized this variant (Fig. 3h) and that the Fu2 dimer and Fu2-Fc again improved neutralization efficiency. The IC_50_ values were even slightly better than when using the Swedish isolate, but the difference is within the intraassay variability. Ty1 failed to neutralize this virus, and the Fu2-Ty1 heterodimer did not neutralize better than Fu2 alone. The beta variant harbors four amino-acid substitutions in the RBD at position K417N, N439K, E484K, and N501Y. Only N501 and E484 are in proximity of the Fu2 interaction interface. In silico analyses with the PISA server^28^ confirmed that the two interfaces do not change in these mutants. Both buried surface areas of the interfaces as well as the number of possible hydrogen bonds remain unchanged (Fig. 3f). These results and analysis combined suggest that Fu2 likely also neutralizes the recently emerged variants gamma (K417T, E484K, N501Y) and alpha (N501Y).

### Therapeutic efficacy of Fu2 in a mouse model of SARS-CoV-2 infection

**Fig. 4:**
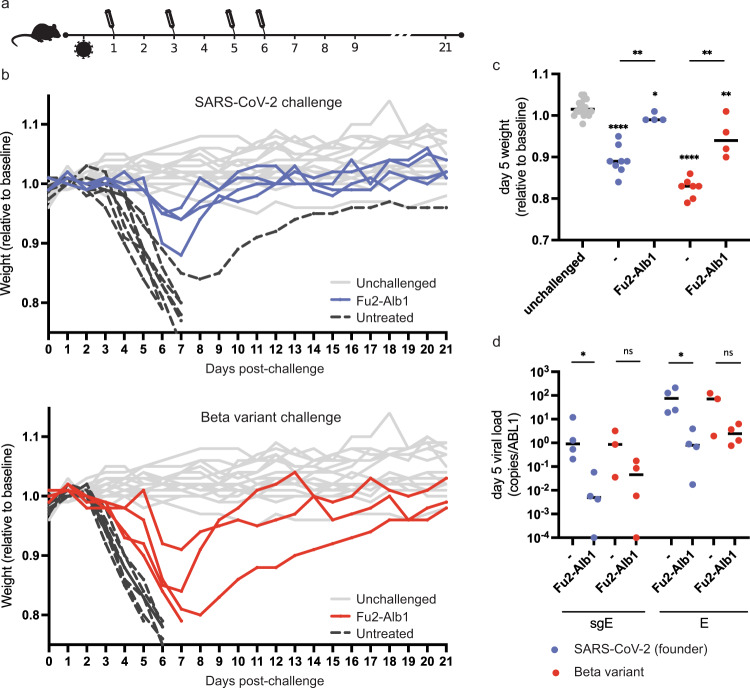
Therapeutically administered nanobody rescues mice from lethal SARS-CoV-2 infection. **a** Timeline of the challenge experiment. K18-hACE2 transgenic mice were challenged with 86 PFU of SARS-CoV-2 (blue, ‘wild-type’) or 100 PFU of the beta variant (red) and injected with Fu2-Alb1 (half-life extended Fu2) at indicated timepoints. **b** Weight of mice during the challenge experiment. The mean weight of each mouse of day 0 to day 2 served as baseline and the weight loss relative this baseline is shown. Uninfected mice (*n* = 18) are shown in gray, untreated infected mice (*n* = 15) in black. Half of the untreated mice are historical controls from identical, previously conducted experiments. The weight for the same unchallenged control mice is shown in the upper and lower panel. **c** Weight loss for day 5 for each group. Fu2-Alb1-treated mice (*n* = 4 animals) experienced significantly reduced weight loss compared to untreated mice (*n* = 7 animals) following infection with wild-type virus (*p* = 0.002; Mann–Whitney U = 0, one-tailed). Significant protection against weight loss was similarly evident in Fu2-Alb1 treated mice (*n* = 4 animals) compared to untreated mice (*n* = 8 animals) following infection with the beta variant (*p* = 0.003; Mann–Whitney U = 0, one-tailed). **d** Analysis of viral load in oropharyngeal samples from mice at day 5 in infected groups. Following infection with wild-type virus, Fu2-Alb1-treated mice (*n* = 4 animals) had significantly reduced viral loads compared to untreated mice (*n* = 7 animals), evident for both E-gene and subgenomic E transcripts expressed as a ratio of copies to copies of ABL1 (*p* = 0.0143, Mann–Whitney U = 0, one-tailed). Statistical comparisons are summarized as: *p ≤ 0.05; **p ≤ 0.01; ****p ≤ 0.0001; ns not significant. Groups displaying significant weight loss compared to uninfected mice are annotated above the points for that group. Exact *P*-values are provided in Table S3. No adjustments were made for multiple comparisons. Source data are provided as a Source Data file for **b** and **d**.

An important requirement for the development of antivirals is neutralization potential in vivo. To address this, we used K18-hACE2 transgenic mice that express human ACE2 as a model for SARS-CoV-2 infection. To determine if Fu2 could reduce disease severity when administered in early infection, we tested its ability to neutralize the virus and protect mice from signs of disease in vivo (Fig. 4). When the Fu2-Ty1 heterodimer was administered prophylactically or therapeutically, we noted that it slightly delayed the onset of disease (Fig. S9). A major issue with using nanobodies therapeutically is their short half-life in vivo. To extend serum half-life, we fused Fu2 to the nanobody Alb1 that binds mouse serum albumin^29^. Mice were challenged with a standardized dose (2.4 × 10^6^ RNA copies) of either a Swedish isolate of SARS-CoV-2 (‘founder virus’, 86 plaque-forming units, PFU) or the beta variant (100 PFU). On days 1, 3, 5, and 6 post infection, mice were injected intraperitoneally (i.p.) with 600 µg of the Fu2-Alb1 heterodimer (Fig. 4a). All infected, untreated mice experienced considerable weight loss within the first week of infection, with mice infected with the beta variant experiencing a slightly faster onset of weight loss. In contrast, treatment with Fu2-Alb1 delayed disease onset and protected mice from severe weight loss following infection with either the founder virus or the beta variant (Fig. 4b, c). Quantification of genomic and subgenomic SARS-CoV-2 E-gene at day 5 also demonstrated that Fu2-Alb1 reduced viral loads, most clearly in mice infected with founder virus (Fig. 4d). Thus, Fu2 is a suitable candidate for antiviral therapy against SARS-CoV-2 and common variants.

## Discussion

Nanobodies represent a valuable alternative to monoclonal antibodies for virus neutralization^30,31^. Their ease of production, thermal stability, and robust biochemical behavior, combined with straight-forward functionalization and multimerization possibilities, make them suitable candidates for therapeutic applications.

The newly emerged SARS-CoV-2 beta variant shows nine amino-acid changes in the spike protein. While biological properties of this variant remain to be determined, two amino-acid substitutions in the receptor binding motif are of potential concern for vaccine efficacy, neutralization efficiency of existing monoclonal antibody therapies, and altered dynamics of virus spread^32–34^. The nanobody described here, Fu2, can effectively neutralize not only the beta variant, but also the more recently dominating delta variant, and therefore has potential to form the basis for development of therapeutic interventions.

Fu2 is also capable of neutralizing SARS-CoV, highlighting its specificity for a more conserved epitope. However, the potency of the monomeric nanobody was significantly reduced, likely because of decreased affinity to the RBD of this virus. That the dimeric constructs significantly increased potency further supports this hypothesis. A similar observation was made for VHH72 originally developed against SARS-CoV, but with some neutralization potential in dimeric form against SARS-CoV-2^26,35^.

Nanobodies typically have short serum half-lives, ranging from 1–3 h^36^, compared to 6–8 days of IgG1^37^. This characteristic can dramatically influence applicability for virus neutralization. Accordingly, for a Fu2-Ty1 heterodimer, we could not detect any protective effect when administered prophylactically, while therapeutic administration significantly delayed disease onset and severity (Fig. S9). In contrast, a half-life extended Fu2-Alb1 heterodimer showed therapeutic efficacy against ‘wild-type’ and the beta variant (Fig. 4), suggesting that nanobody-based constructs have the potential for SARS-CoV-2 antiviral therapy.

In addition to sterically blocking ACE2 interaction, the RBD-specific nanobody Fu2 rapidly induces the formation of spike trimer–dimers and leads to aggregation of virions. While we cannot determine the precise contribution of the aggregation to overall neutralization, we expect that it adds an additional layer of protection, especially in situations with high local virion and/or Fu2 concentration. Based on our data, we hypothesize that the nanobody first engages the RBD at the conserved ‘interface major’, which is the strong interaction that is measured by SPR (Fig. 1d). The RBD of Fu2-bound spike is positioned in the ‘up’ conformation, which permits engaging another Fu2-bound spike trimer. The interaction at the interface-minor is substantially weaker but compensated for by the avidity effects of the 6 nanobodies of the complex. In contrast to spike trimer–dimers reported for the full-length spike that are based on S1–S1 interactions placing the trimers ‘side-by-side’^38^, Fu2 induced a dimer in a ‘head-to-head’ orientation with the C-termini at the distal ends. Targeting two epitopes increases the binding surface area, but, more importantly, when locked in such a conformation the function of each spike is sterically blocked both by the nanobody and by the other spike in such a way that the whole complex must dissociate for the spike to regain its function. Interestingly, another study reported a nanobody that binds to similar epitope as Fu2 and induces elongated structure with two copies of the spike with all RBDs in the up conformations, but they were unable to define the structural basis for the observed phenomenon^39^.

The interaction between Fu2 and the RBD is highly unusual, and for a monomer to induce antigen dimerization in this manner, two copies of an antigen must be bound at different epitopes with precisely the right spatial arrangement. This mode of antigen binding may be uncommon in naturally elicited antibody repertoires but could be achievable by structure-based antibody design and might present a general strategy for potent immobilization of viral glycoproteins and other antigens. It remains to be seen whether ‘designer’ bispecific monomer nanobodies can push the potency envelope beyond what is currently attainable.

## Methods

### Cells and viruses

HEK293T cell (ATCC-CRL-3216 and ATCC-CRL-11268) and Vero E6 cells (ATCC-CRL-1586) were maintained in DMEM (Gibco) supplemented with 10% fetal calf serum and 1% penicillin-streptomycin in a humidified incubator with 5% CO_2_ at 37 °C. Calu-3 cells were additionally supplemented with nutrient mixture F12 (Thermo Fisher Scientific). The HEK293T cell line was further engineered to overexpress human ACE2 by lentiviral transduction^14^. All cell lines used for the experiments were negative for mycoplasma as determined by PCR.

Infectious SARS-CoV-2 (GenBank accession number MT093571)^27^ and the beta variant^1^ isolate used for PRNTs and the challenge experiment in Fig. S9 were propagated in Vero E6 cells and titrated by plaque assay. Viruses used for the challenge in Fig. 4 were propagated in Calu-3 cells, quantified by qPCR, and titrated by plaque assay in Vero E6 cells. Challenge stocks for experiments shown in Fig. 4 were confirmed by Sanger sequencing to harbor no high frequency cell culture adaptation mutations in spike. For negative-stain electron microscopy, viral particles were concentrated by centrifugation through a 20% sucrose cushion at 80,000 × g for 1.5 h at 4 °C (Sorvall wx80, rotor TH-641). Pellets were resuspended in PBS.

### Proteins and probes

The plasmid for the expression of the SARS-CoV-2 prefusion-stabilized spike (2 P) ectodomain was a kind gift from the McLellan lab^6^. The plasmid was used for transient transfection of FreeStyle 293 F cells using the FreeStyle MAX reagent (Thermo Fisher Scientific). The spike trimer was purified from filtered supernatant on Streptactin XT resin (IBA Lifesciences) or Ni-NTA resin and purified by size-exclusion chromatography on a Superdex 200 (Cytiva). For cryo-EM a similar construct with 6 proline substitutions (S6-P) was used^40^, and the protein was purified on His-Pur Ni-NTA resin (Thermo Fisher Scientific) followed by size-exclusion chromatography. The RBD domain was cloned upstream of a sortase A motif (LPETG) and a 6xHIS tag. The plasmid was used for transient transfection of FreeStyle 293 F cells as described above. The protein was purified from filtered supernatant on His-Pur Ni-NTA resin followed by size-exclusion chromatography on a Superdex 200. The albumin binding nanobody Alb1 was described earlier^29^ and the sequence was obtained from WO/2006/122787. Nanobodies were cloned in the pHEN plasmid with a C-terminal sortase motif (LPETG) and a 6xHIS tag. BL21 *E. coli* were transformed with this plasmid, and expression was induced with 1 mM IPTG at OD600 = 0.6 and cells were grown overnight at 30 °C. Nanobodies were retrieved from the periplasm by osmotic shock and purified on Ni-NTA resin and size-exclusion chromatography.

For nanobody-Fc fusions, the nanobody sequence was cloned upstream of a human IgG1-Fc and a hinge region (SDKTHTCPPCP). The plasmid was used to transiently transfect FreeStyle 293 F cells using the Freestyle MAX reagent. The nanobody-Fc fusion was purified on Protein G Sepharose and by size-exclusion chromatography.

Sortase A 5 M was produced as described before in BL21 *E. coli* and purified by Ni-NTA and size-exclusion chromatography^3^. Fluorescent spike ectodomain was generated by first attaching dibenzocyclooctyine-*N*-hydroxysuccinimidyl ester (DBCO-NHS) to the spike trimer in a 3:1 molar ratio, before attaching AbberiorStar-635P-azide by click-chemistry. The final product was purified from unreacted DBCO and fluorophore on a PD-10 desalting column. The biotinylated RBD was generated using sortase A and amine-PEG3-biotin as a nucleophile. The reaction was performed with 50 µM RBD, 5 µM sortase A 5 M, and 8 mM amine-PEG3-biotin in 50 mM Tris pH 7.5, 150 mM NaCl, for 6 h at 4 °C. Sortase A and unreacted RBD was removed on Ni-NTA resin and excess nucleophile was removed by two consecutive purifications on PD-10 desalting columns.

### Alpaca immunization, library generation, and nanobody isolation

One adult female alpaca (*Funny*) at PreClinics, Germany, was immunized four times in a 60-day immunization schedule. Each immunization consisted of two subcutaneous injections behind the shoulder blade on the right and left flank using the GERBU Fama adjuvant (GERBU Biotechnik Gmbh). For the first immunization, 200 µg of prefusion-stabilized spike and 200 µg of S1 + S2 domain (Sino Biologicals) was used. Remaining immunizations each consisted of one injection with 200 µg RBD and one injection with 200 µg prefusion-stabilized spike, both produced in Freestyle 293 F cells as described above. Serum from the final bleed at day 60 neutralized SARS-CoV-2 pseudoviruses with an ID50 > 20 000. The animal study protocol was approved by the PreClinics animal welfare officer commissioner and registered under the registration No. 33.19-42502-05-17A210 at the Lower Saxony State Office for Consumer Protection and Food Safety—LAVES and is compliant with the Directive 2010/63/EU on animal welfare. The nanobody phage library was generated as described^14^ (library size 7.2 × 10^7^). Two consecutive rounds of phage selection were performed on spike immobilized on magnetic beads. In the first round, we used 200 µl of phage and 20 µg of antigen, in the second round 2 µl of phage and 2 µg of antigen. In an ELISA-based binding screen, one clone showed the strongest response to both RBD and spike and was characterized in detail for this paper. The same library has been more extensively characterized in another manuscript^41^.

### Dimer generation using click-chemistry

To generate homo- and heterodimers, the different nanobodies were first site-specifically functionalized on the C-terminus using sortase A with either an azide or a dibenzocyclooctine (DBCO) as described in detail here33. In brief, for functionalization with DBCO, 70 µM of nanobody was incubated with 5 µM sortase A, 8 mM DBCO-amine (Sigma–Aldrich) in 150 mM NaCl, 50 mM Tris pH 7.5 and 10 mM CaCl_2_ for 3 h at 25 °C. To functionalize the nanobody with an azide, 70 µM of nanobody, was incubated with 5 µM sortase A 5 M, 10 mM 3-azido-1-propanamine (Sigma–Aldrich #762016) in 150 mM NaCl, 50 mM Tris pH 7.5 and 10 mM CaCl_2_ for 3 h at 25 °C. In both reactions, unreacted nanobody, sortase A and excess nucleophile were removed using Ni-NTA resin and PD-10 columns or size-exclusion chromatography. The click reaction was initiated by mixing azide and DBCO labeled nanobody in a 1:1 molar ratio for 48 h at 4 °C. Dimers were purified from unreacted monomeric nanobody by size-exclusion chromatography on a Superdex S200 16/600 column (Cytiva).

### Neutralization assays

Pseudotyped virus neutralization assay: Pseudotyped viruses were generated by co-transfection of HEK293T cells with plasmids encoding firefly luciferase, a lentiviral packaging plasmid (Addgene cat#8455), and a plasmid encoding either the SARS-CoV or SARS-CoV-2 spike protein (founder strain^42^, beta^43,44^, or delta^44^) harboring a C-terminal truncation of 18 amino acids. Media was changed 12–16 h post-transfection, and pseudotyped viruses were harvested at 48 and 72 h, filtered through a 0.45 µm filter and stored at −80 °C until use. Pseudotyped viruses sufficient to generate 100,000 relative light units (RLU) were incubated with serial dilutions of nanobody for 60 min at 37 °C. 15,000 HEK293T-ACE2 cells were then added to each well, and the plates were incubated for 48 h at 37 °C. Luminescence was measured using Bright-Glo (Promega) on a GM-2000 luminometer (Promega) with an integration time of 0.3 s.

Plaque reduction neutralization test: One day before the experiment, 100,000 Vero E6 cells were seeded per well of a 24-well plate. A 3-fold serial dilution of the nanobody constructs starting at 10 µg/ml were incubated with 100 plaque-forming units (PFU) of the first Swedish clinical isolate of SARS-CoV-2 or beta variant for 1 h at 37 °C. The nanobody-virus mixture was then added to the cells and incubated at 37 °C for 1 h. Cells were washed with PBS, and an overlay of 1% CMC/DMEM supplemented with 2% FBS was pipetted into the wells. Three days post infection, cells were fixed in 10% formaldehyde, followed by staining with crystal violet. Plaques were counted and inhibition determined by comparing to control wells on the same plate.

### Flow cytometry

HEK293T-hACE2 cells were trypsinized and fixed in 4% formaldehyde in PBS for 20 min. Cells were stained with spike-AbberiorStar-635P not premixed or premixed with Fu2 or control nanobody. Fluorescence was quantified using a BD FACSCelesta and the FlowJo software package.

### Surface plasmon resonance

Binding kinetics were determined by surface plasmon resonance using a Biacore 2000. All experiments were performed at 25 °C in a running buffer of 10 mM HEPES, 150 mM NaCl, pH 7.4, and 0.005% Tween-20 (v/v). Site-specifically biotinylated RBD was immobilized on streptavidin sensor chips (Series S sensor Chip SA, GE Healthcare) to a level of ~200 resonance units (RU). A 2-fold dilution series of the nanobodies was injected at a flow rate of 30 µl/min (association 180 s, dissociation 900 s), and the immobilized RBD was regenerated using 0.1 M glycine-HCL buffer pH 2 for 2 × 10 s. Data were analyzed using BIAevaluation Software and fitted using the 1:1 Langmuir model with mass transfer.

### Next-generation sequencing

Next-generation sequencing on pre- and post-enrichment nanobody libraries was performed as described in Hanke et al. 2020^14^ on an Illumina MiSeq.

### Gel-filtration shift assays

For the gel-filtration shift assay, 80 µg of prefusion-stabilized spike protein was incubated with or without nanobody for 15 min and was run on a Superose 6 increase 10/300 GL (Cytiva), and absorbance at 280 nm was detected. Sample and injection volumes were kept identical between runs to obtain comparable elution profiles.

### Fluorescent pseudotyped lentivirus brightness measurements

To generate fluorescent lentiviral particles enveloped with the spike protein of the SARS-CoV-2 coronavirus (PSV-GFP), HEK 293 T cells were reverse-transfected with 3 plasmids: psPAX2 (a gift from Didier Trono, Addgene #12260), pEGFP-Vpr (obtained through the NIH HIV Reagent Program, Division of AIDS, NIAID, NIH: pEGFP-Vpr, ARP-11386, contributed by Dr. Warner C. Greene) and pCMV14-3X-Flag-SARS-CoV-2 S (a gift from Zhaohui Qian, Addgene #145780) at 1:0.5:2 molar ratio using Lipofectamine™ 2000 (0.6 ul per 1 ug DNA; Invitrogen™, 11668019); growth medium was refreshed 16 h after transfection. Virus-rich supernatant was collected twice over the next 48 h and, if needed, stored at 4 °C prior to the concentration. Supernatants were pooled, mixed with the Lenti-X™ Concentrator (Takara Bio, 631231) at 3:1 ratio (v:v) and incubated overnight at 4 °C. The solution was then spun down at 1500 × g for 45 min at RT, the pellet was resuspended in sterile 1× PBS (Sigma–Aldrich, D8537) and stored at −80 °C.

PSV-GFP was incubated with 100 µg/ml Ty1, Fu2, or PBS as control for 1 h. The mixtures were transferred to an eight-well glass bottom Ibidi chamber. Fluorescence Correlation Spectroscopy (FCS) measurements were performed using Zeiss LSM 780 microscope. Four hundred eighty eight nanometers argon ion laser was used to excite GFP. ×40/1.2 NA water immersion objective was used to focus the light in solution. Forty to fifty curves were taken (5 s each) in solution where PSV-GFP particles diffuse freely. Laser power was set to 0.1% of the total laser power that corresponds to ≈2 µW. Curves were then fitted with FoCuS-point software^45^ to extract number of diffusing species and brightness of single diffusing species.

### Negative-stain electron microscopy

Concentrated SARS-CoV-2 was incubated in the absence or presence of 20 µg/ml of Fu2 for 1 h under agitation. Samples were fixed in EM-grade paraformaldehyde (Ted Pella) and used to prepare negative staining grids. Four microliters of sample was loaded on the grids (EM Resolutions (EMR), 200 squares, carbon support film on copper) that had been glow-discharged with 25 mA for 1 min using an EMS ×100 (Electron Microscopy Sciences) glow-discharge unit. The samples were incubated for 4 min on the grids and subsequently stained with 1% uranyl acetate solution (Ted Pella). The grids were imaged in Talos 120 C G2 (Thermo Scientific) equipped with a CETA-D detector in the Karolinska Institutet’s 3D-EM facility (https://ki.se/cmb/3d-em). Micrographs were collected using Serial-EM^46^ at a magnification of ×45,000, corresponding to a pixel size of 3.14 Å/pixel.

### Cryo-EM sample preparation and imaging

Spike trimer (1.8 mg/ml) S6-P and Fu2 (2.1 mg/ml) were mixed in a 1:6 molar ratio followed by incubation on ice for 10 min. Prior to cryo-EM grid preparation, grids were glow-discharged with 25 mA for 2 min using an EMS ×100 (Electron Microscopy Sciences) glow-discharge unit. CryoMatrix® holey grids with amorphous alloy film (R 2/1 geometry; Zhenjiang Lehua Technology Co., Ltd) were used. Three microliters aliquots of sample solutions were applied to the grids and the grids with sample were then vitrified in a Vitrobot Mk IV (Thermo Fisher Scientific) at 4 °C and 100% humidity (blot 10 s, blot force 3595 filter papers (Ted Pella Inc.)). Cryo-EM data collection was performed with EPU (Thermo Fisher Scientific) using a Krios G3i transmission-electron microscope (Thermo Fisher Scientific) operated at 300 kV in the Karolinska Institutet’s 3D-EM facility (https://ki.se/cmb/3d-em). Images were acquired in 165 kx nanoprobe EFTEM SA mode with a slit width of 10 eV using a K3 Bioquantum. Exposure time was 1.5 s during which 60 movie frames were collected with a fluency of 0.81 e-/Å2 per frame (Table S1). Motion correction, CTF-estimation, Fourier binning (to 1.01 Å/px), picking and extraction in 600-pixel boxes (size threshold 400 Å, distance threshold 100 Å, using the pretrained BoxNet2Mask_20180918 model) were performed on the fly using Warp^47^. Due to preferential orientation, we collected a part of the dataset with a 25-degree stage tilt.

A total of 14,081 micrographs were selected based on an estimated resolution cutoff of 4 Å and defocus below 2 microns as estimated by Warp. Furthermore, Warp picked 1,035,962 particles (dimer of trimeric spike with Fu2) based on above-mentioned criteria. Extracted particles were imported into cryoSPARC v3.1^48^ for 2D classification, 3D classification and nonuniform 3D refinement. After 2D classification, clean classes with high-resolution features (and with characteristic trimeric spike views) were retained and used to generate ab initio 3D reconstructions. In total 277,372 particles were retained for refinement steps and these particles were processed with C1 symmetry (image processing scheme Fig. S7).

These particles were further processed using heterogeneous refinement that resulted in a reconstruction with high-resolution structural features in the core of the spike. One round of homogeneous refinement was followed by nonuniform refinement. All final reconstructions were analyzed using 3D-FSC^49^ and although there was moderate anisotropy in the full map reconstructions, the localized reconstruction displayed no significant anisotropy (Fig. S8A, B). All CTF refinements were per-particle CTF refinements interspersed with global aberration correction (beamtilt, trefoil, tetrafoil and spherical aberration). Please see Table S1 for data collection and processing statistics and the respective cryo-EM data processing schemes. For the Spike-Fu2-dimer interface we used a particle set with partial-signal subtraction of all parts except for Fu2-RBD dimer interface (containing two RBDs and two Fu2). From this we performed local reconstruction (nonuniform). The local reconstruction resulted in a map with 2.89 Å overall resolution as compared to 3.2 Å overall resolution for the full map.

### Cryo-EM model building and structure refinement

The structure of the spike protein trimer PDB: 7KSG^5^ was used as a starting model for model building. A homology model for Fu2 was generated by SWISS-MODEL^50^ with PDB: 5LHN as template (chain B)^51^. Structure refinement and manual model building were performed using Coot^52^ and PHENIX^53^ in interspersed cycles with secondary structure, Ramachandran, rotamers, and bond geometry restrains. Structure figures and EM density-map figures were generated with UCSF ChimeraX^54^ and COOT, respectively. Please see Table S2 for refinement and validation statistics.

### SARS-CoV-2 challenge experiments

K18-hACE2 transgenic mice were purchased from Jackson laboratories and maintained as a hemizygous line. Experiments were conducted in BSL3 facilities at the Comparative Medicine department (KM-F) at Karolinska Institutet. Ethics for studies of virus infection and therapeutic intervention were obtained from the Swedish Board of Agriculture (10513-2020). Mice were housed in individually ventilated cages with 12 h/12 h light/dark cycles and had access to food and water ad libitum. Cage enrichment included shredded cardboard and paper rolls. Cage and water changes were performed on a weekly basis and general monitoring of all mice was performed daily by technical staff. Mice were administered nanobodies as described in the main text and challenged intranasally with 86 PFU SARS-CoV-2 (‘wild-type’, Swedish isolate) or 100 PFU the beta variant in 40 µl PBS following isoflurane sedation. The challenge in Fig. S9 was performed with 1000 PFU of SARS-CoV-2 (Swedish isolate) propagated in Vero E6 cells. Oropharyngeal sampling was performed at the indicated timepoints under light anesthesia with isoflurane. Weight and general body condition were monitored daily until weight drop started, whereupon mice were monitored twice daily. During the experiment, weight loss, changes in general health, breathing, body movement and posture, piloerection, and eye health were monitored. Mice were typically sacrificed when they achieved 20% weight loss. However, some mice were sacrificed before losing 20% body weight, when movement was greatly impaired and/or they had breathing difficulty considered to reach a severity level of 0.5 on Karolinska Institutet’s veterinary plan for monitoring animal health. The weight loss in response to infection was highly reproducible. In Fig. 4 data from 50% of the challenged and untreated animals are historical controls from previous experiments performed under identical conditions. Statistical analyses were two-sided Mann–Whitney tests, implemented in Prism 9.

### RNA Extraction and RT-qPCR for SARS-CoV-2 detection

Viral RNA was isolated from buccal swabs collected on dpi 6 and stored in 500 µl of TRIzol™ Reagent (Invitrogen). Total RNA extractions from buccal swab samples were performed using an adapted TRIzol™ manufacturers protocol with a 45 min precipitation step at −20 °C. RNA pellets were resuspended in 20 µl of RNase-free water.

RT-PCR reactions were performed using 4 µl of resuspended RNA in a 20 µl reaction volume using the Superscript III one step RT-qPCR system with Platinum Taq Polymerase (Invitrogen) with 400 nM concentrations of each primer and 200 nM of probe. Primers and probes for the CoV-E-gene target were as previously described^55^. Primers and probes for the ABL1 target were adapted from Ishige et al.^56^ to enable detection of the murine homolog: (ABL1_ENF1003_deg: 5′-TGGAGATAACACTCTCAGCATKACTAAAGGT-3′, ABL1_ENR1063: 5′-GATGTAGTTGCTTGGGACCCA-3′, ABL1_ENPr1043_deg: 5′-HEX-CCATTTTTSGTTTGGGCTTCACACCATT-BHQ1-3′). The CoV-E and ABL1 TaqMan assays were run in multiplex. Detection of the subgenomic CoV-E target was adapted from Wölfel et al.^57^, using a leader/E-gene junction specific forward primer: (sgEjunc_SARSCoV2_F: 5′-CGATCTCTTGTAGATCTGTTCTCTAAACG-3′). All oligonucleotides were synthesized by Eurofins Genomics.

Thermal cycling conditions for all assays consisted of RT at 55 °C for 10 min, denaturation at 95 °C for 3 min, and 45 cycles of 95 °C, 15 s and 58 °C, 30 s. Reactions were carried out using a CFX96 Connect Real-Time PCR Detection System (Bio-Rad) following manufacturer instructions. To generate standard curves, a synthetic DNA template gBlock (Integrated DNA Technologies) was transcribed using the mMessage mMachine™ T7 Transcription Kit (Invitrogen) and serially diluted. To reduce sampling-related variability, SARS-CoV-2 RNA copies were normalized by ABL1 copies, and this ratio was used for comparisons. ABL1 copies were not significantly different between groups^21,58,59^.

### Reporting summary

Further information on research design is available in the Nature Research Reporting Summary linked to this article.

## Supplementary information

Supplementary Information

Reporting Summary

## Data Availability

The cryo-EM density maps have been deposited in the Electron Microscopy Data Bank under accession codes EMD-12561 (dimer of spike trimer + 6 Fu2) and EMD-12465 (localized reconstruction of 2 RBDs + 2 Fu2). The atomic coordinates have been deposited in the Protein Data Bank under IDs 7NS6 (dimers of spike trimers + 6 Fu2) and 7NLL (localized reconstruction of 2 RBDs + 2 Fu2). The amino-acid sequence of Fu2 can be obtained under the same accession codes, and the Fu2 variants are shown in Fig S2e. Source data are provided with this paper.

## Source data

Source Data
