# Supplementary material for: A bispecific monomeric nanobody induces spike trimer dimers and neutralizes SARS-CoV-2 in vivo
Source: Nat Commun. 2022 Jan 10;13:155. doi: 10.1038/s41467-021-27610-z (PMC8748511; doi:10.1038/s41467-021-27610-z)
Supplement: Supplementary file 1 — Supplementary Information [file 41467_2021_27610_MOESM1_ESM.pdf]

Supplementary figures and tables

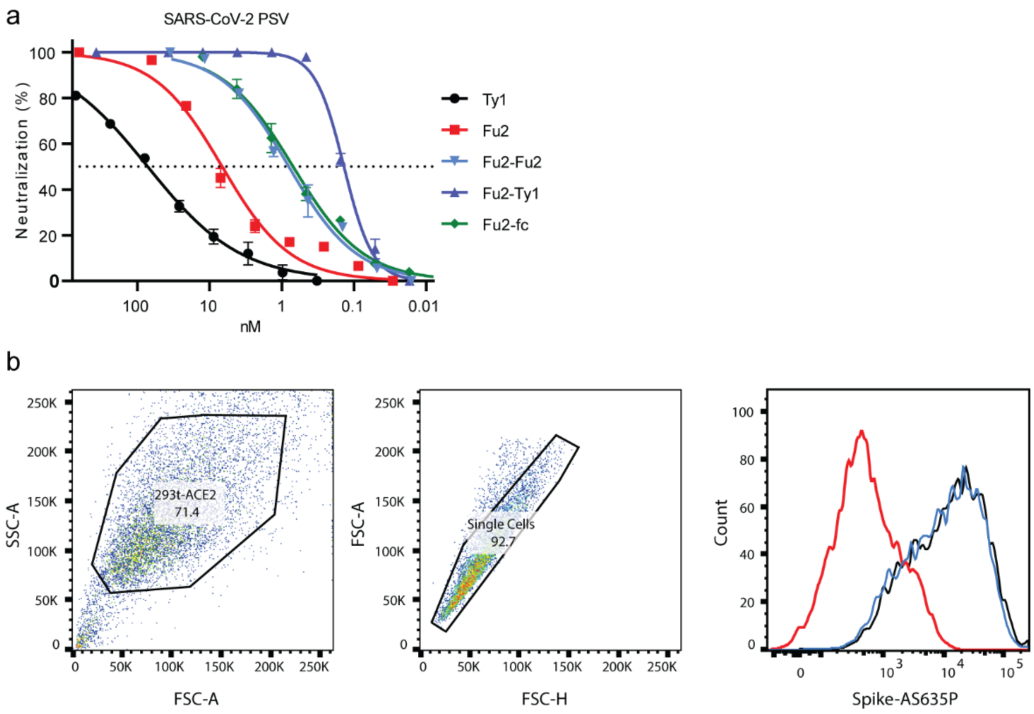

	Sample Name	Subset Name	Count
(blue)	HEK293t-ACE-SPIKE-AS635P-control nb	Single Cells	6599
(red)	HEK293t-ACE-SPIKE-AS635P+Fu2	Single Cells	6543
(black)	HEK293t-ACE-SPIKE-AS635P	Single Cells	6618

Fig. S1. Neutralization of SARS-CoV-2. (a) These data are from the same experiment as presented in Fig. 1b with the distinction that the x-axis is shown nM instead of µg/ml. Data from two (Fu2) or three (all others) replicates is shown, and the error bars represent the mean with standard deviation. (**b**) Gating strategy for Fig. 1g.

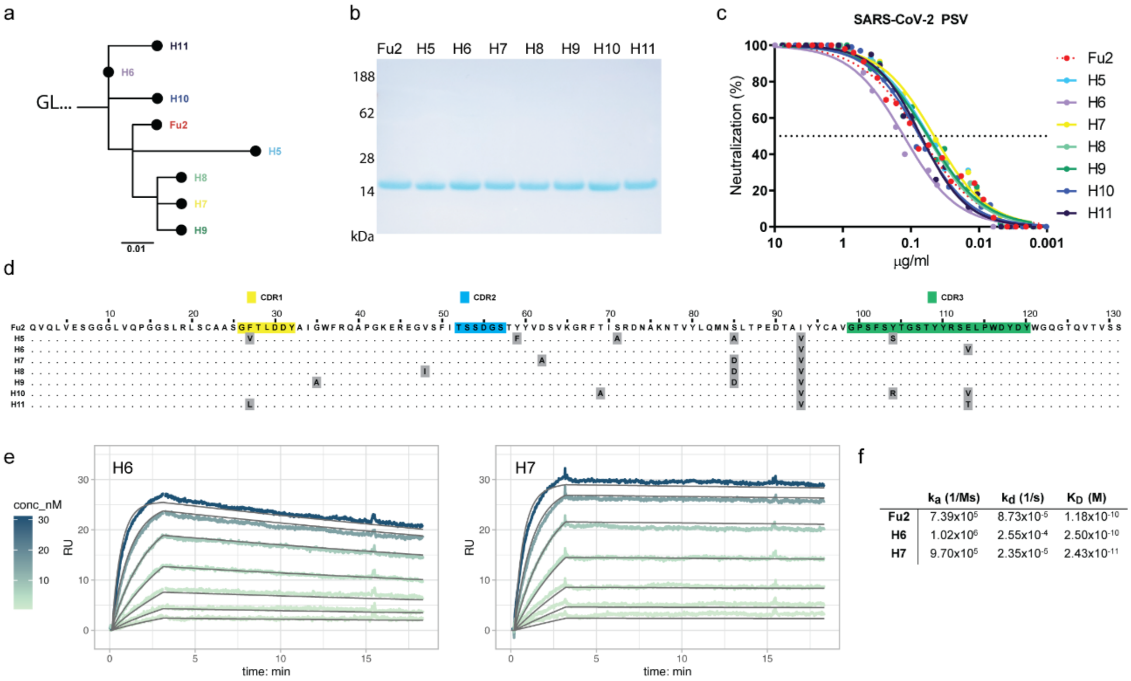

	k_a (1/Ms)	k_d (1/s)	K_D (M)
**Fu2**	7.39×10^{5}	8.73×10^{-5}	1.18×10^{-10}
**H6**	1.02×10^{6}	2.55×10^{-4}	2.50×10^{-10}
**H7**	9.70×10^{5}	2.35×10^{-5}	2.43×10^{-11}

Fig. S2. Fu2 variants identified by next-generation sequencing (NGS) display similar neutralization potential and binding kinetics. (**a**) Average difference of Fu2 variants is shown as a tree diagram. (**b**) Fu2 and seven Fu2-variants were expressed in *E. coli*, purified, and analyzed by SDS-PAGE and Coomassie staining. (**c**) A dilution series of Fu2 and its variants were incubated with SARS-CoV-2 pseudotyped lentivirus (PSV) for 1 hour at 37 °C before infecting HEK293T-hACE2 cells. Neutralization (in %) compared to the untreated PSV is shown. (**d**) Sequences of the Fu2 variants. Amino acid substitutions are indicated, and complementary determining regions (CDRs) are marked. (**e**) Binding kinetics of Fu2 and variants to the RBD were measured by surface plasmon resonance (SPR). Site-specifically biotinylated RBD was immobilized on streptavidin sensor chips, and kinetics for a dilution series of the indicated monomeric nanobodies were measured. Sensorgrams are color-coded based on concentration. The fit is based on the 1:1 Langmuir model and is shown in dark grey solid lines. (**f**) Kinetic parameters of Fu2 and Fu2 variants binding to the RBD.

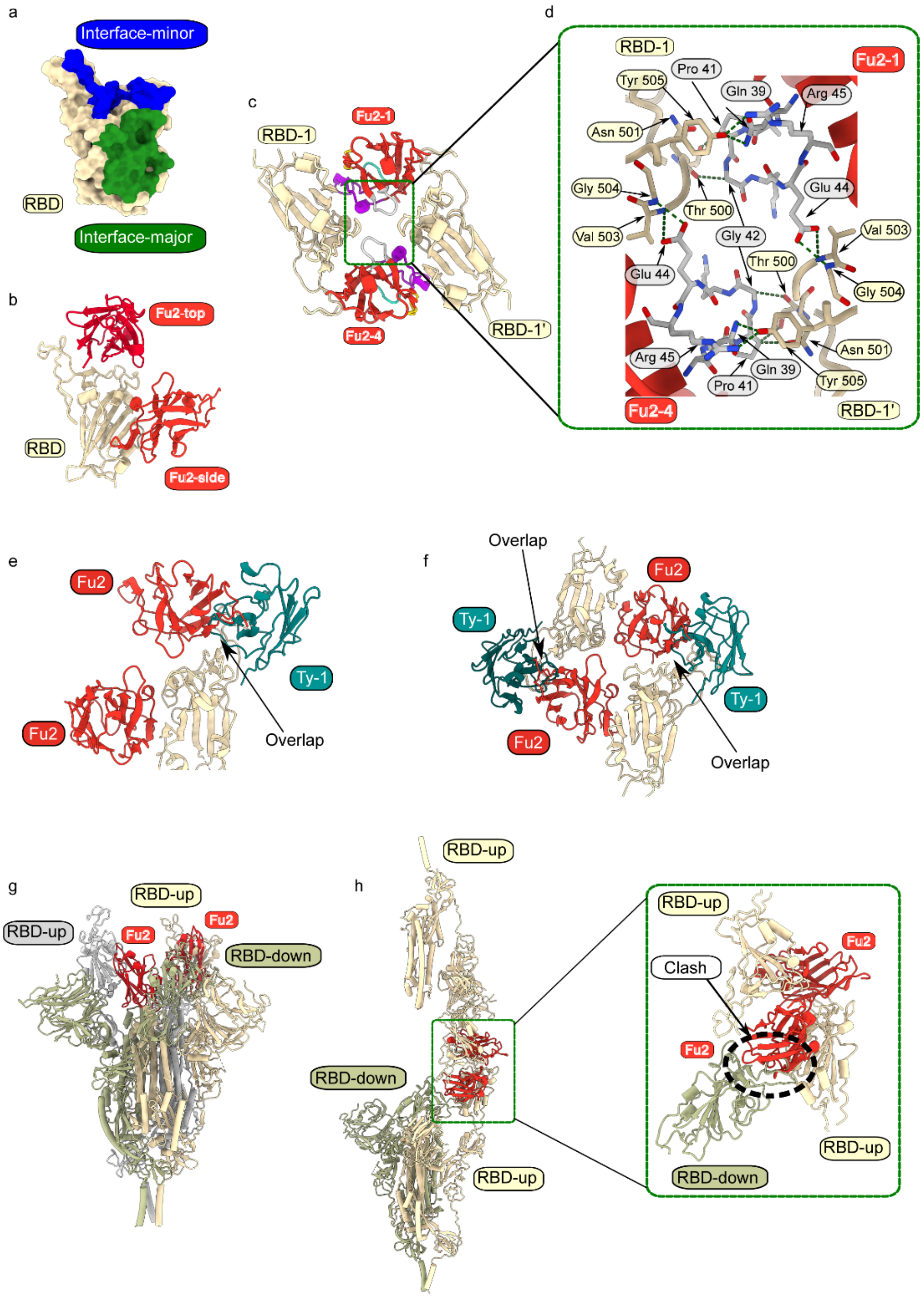

Fig. S3. (**a**) Surface view of the RBD with interface-major and interface-minor color coded. (**b**) Relative positions of two Fu2 molecules binding the RBD. (**c**) The interaction between two RBDs and two Fu2s showing in grey the β-hairpins in the Fu2 framework region (39-45) that help to mediate the dimer-of-trimers Fu2-spike complex. (**d**) Close-up view of (**c**). (**e** and **f**) Assessment of Fu2-Ty1 heterodimer binding. Alignment of the Spike-Fu2 structure to the Spike-Ty1 structure (RBD in 'up' conformation) (PDB:6ZXN[14]). Arrows indicate partial overlap of binding surface on RBD. (**g**) Assessment of Fu2-RBD interaction in RBD-down conformation. RBD-Fu2 structure superimposed to RBD-down of spike in 2-up conformation (PDB:7A29[13]) (**h**) Assessment of Fu2-RBD interaction in RBD-down conformation in dimeric spike. 2-up spike (PDB:7A29[13]) is shown superimposed with the spike dimer-of-trimers.

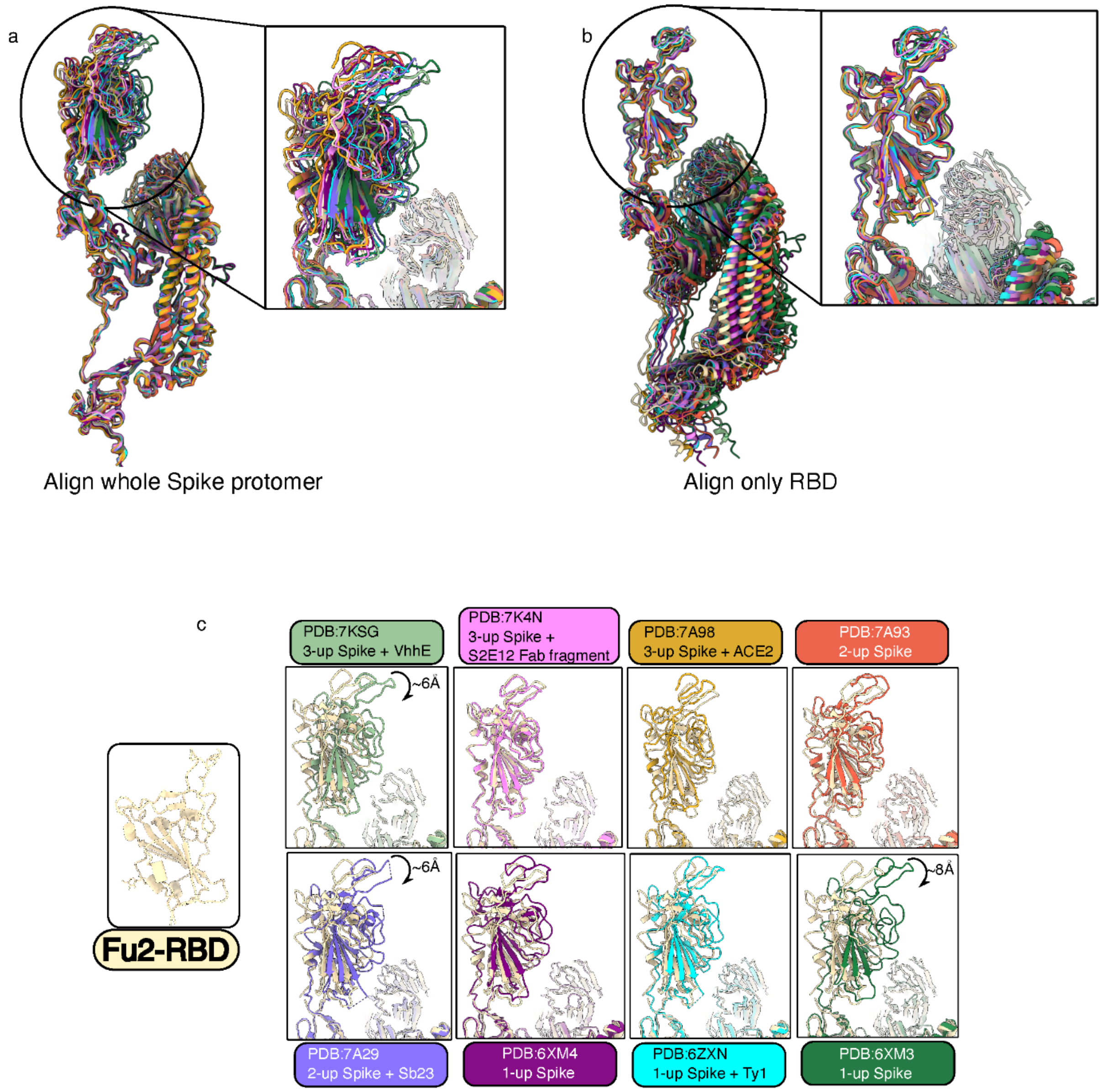

Fig. S4. Assessment of Fu2 mediated structural changes in spike structure. (**a**) Alignment of dimeric spike trimer with spikes in 1-up, 2-up and 3-up conformation (one protomer in 'up' state shown). (**b**) Structural alignment of dimeric spike trimer with RBDs of 1-up, 2-up and 3-up spikes (protomer in 'up' conformation). (**c**) Structural comparison of RBD (Fu2-bound dimeric spike) with eight different RBDs structures (7KSG[5], 7K4N[21], 7A98[58], 7A93[58], 7A29[13], 6XM3[59], 6XM4[59], 6ZXN[14]).

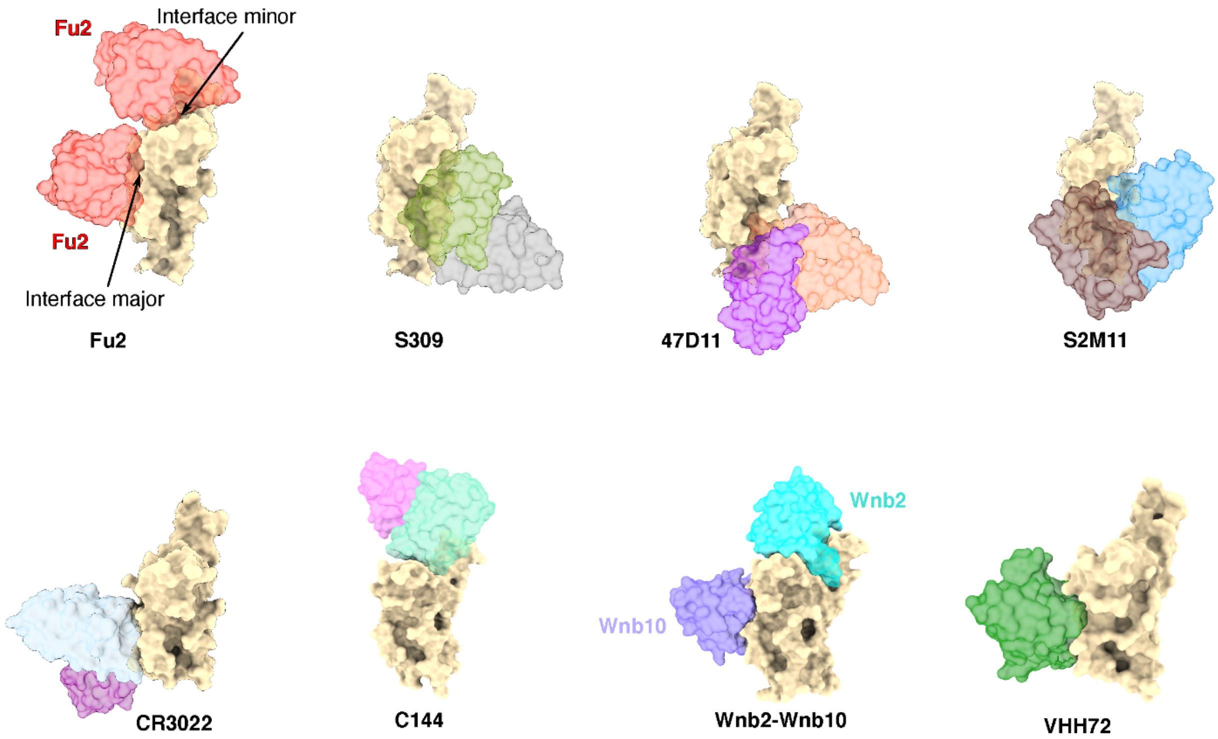

Fig. S5 The binding site of Fu2 in comparison with other antibodies and nanobodies. The binding sites of Fu2 are compared with S309 (Fab fragment)[19], the monoclonal antibodies 47D11[20] (PDB:7AKD) and S2M11[21] (PDB:7K43), CR3022[22] (PDB:7K90), C144[24] (PDB: 7K90) and nanobodies Wnb2 and Wnb10 (PDB:7LX5[25]) and VHH72 structure (PDB:6WAQ[26]).

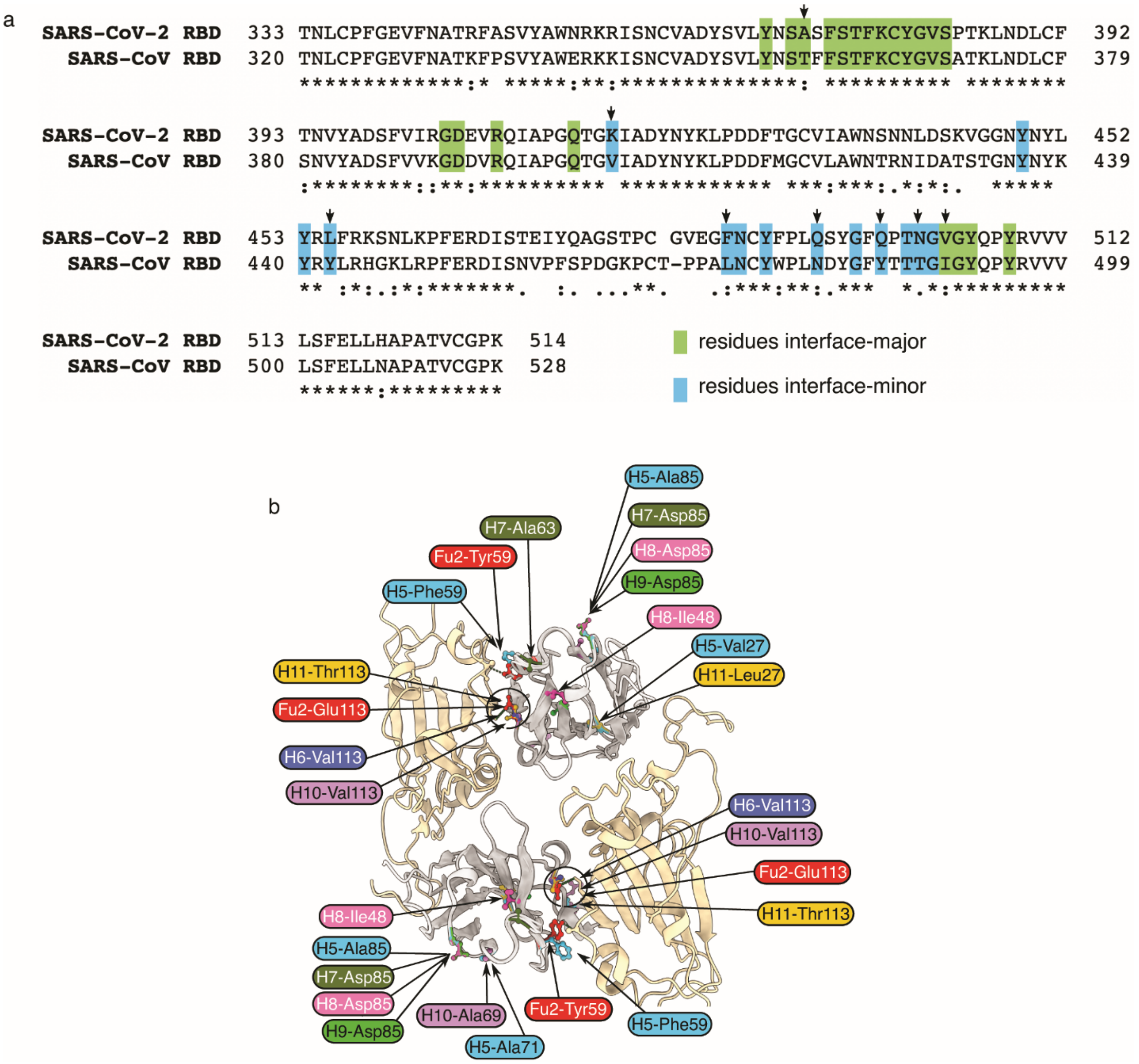

Fig. S6. (**a**) Sequence alignment of RBDs from SARS-CoV and SARS-CoV-2. Interface-major and interface-minor residues are highlighted. Arrows indicate the different amino acids of the interface. (**b**) Fu2 variants and analysis of interface residues. Distinct residues of different variants shown as sticks (color coded).

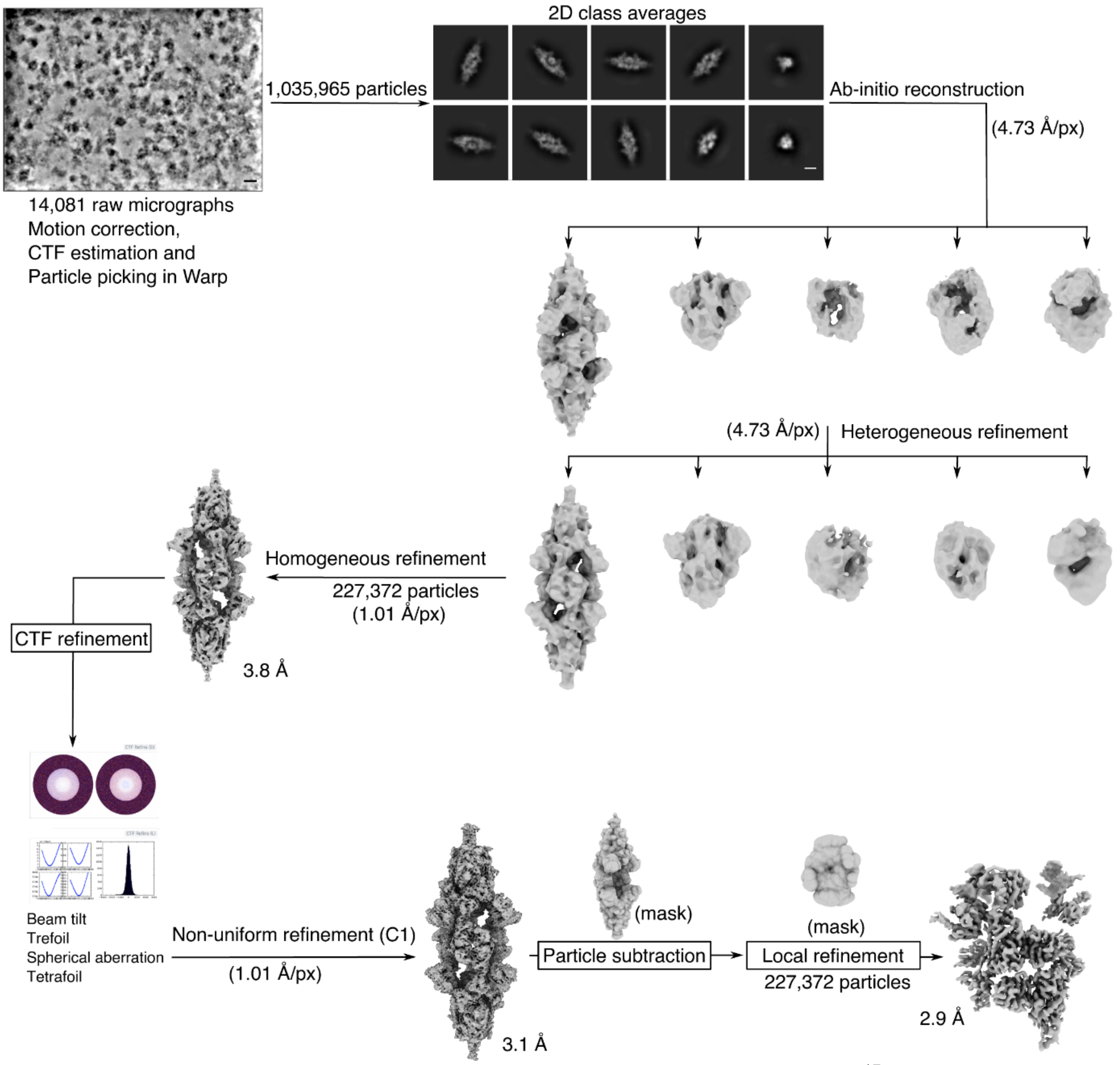

Fig. S7. Cryo-EM image processing scheme. Particles picked by Warp[47] were processed in cryoSPARC v3.1[48]. Representative 2D class averages are shown (scalebar = 100Å). Particles contributing to the clean classes were used to generate ab-initio reconstructions (five classes) followed by heterogeneous refinement. One class showing high-resolution features was refined further (C1 symmetry). For localized reconstruction, particle subtraction followed by local refinement (Non-uniform) was performed.

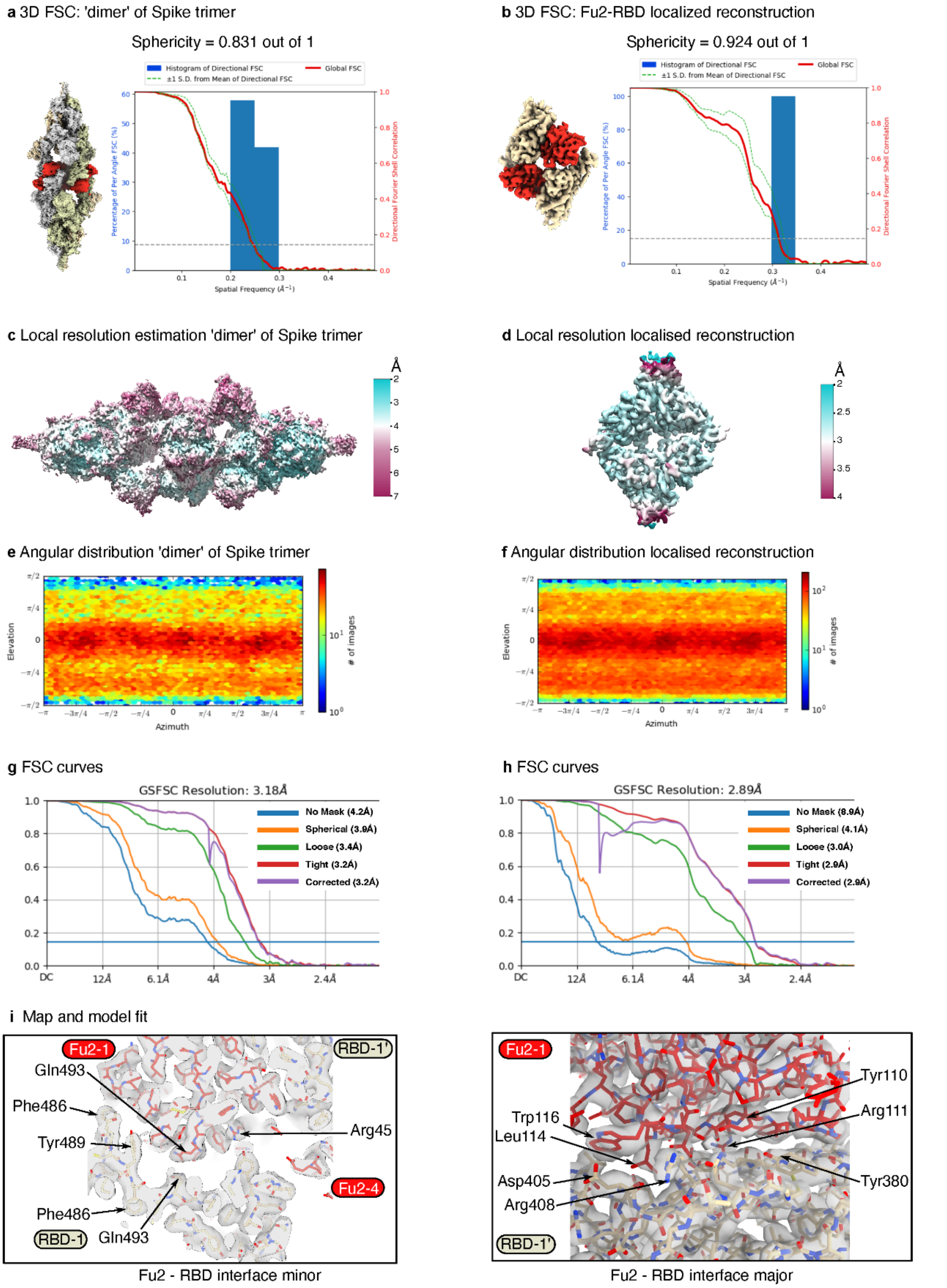

Fig. S8 Cryo-EM validation. (**a**) and (**b**) 3D FSC[51] and sphericity of 'dimer' of spike trimer and of localized map, (**c**) and (**d**) Local resolution estimation of 'dimer' of spike trimer and local resolution estimation of localized map (**e**) and (**f**) Angular distribution of 'dimer' of spike trimer reconstruction and localized reconstruction map (**g**) and (**h**) FSC curves of 'dimer' of spike trimer and of localized map (**i**) model-map fitting atomic resolution.

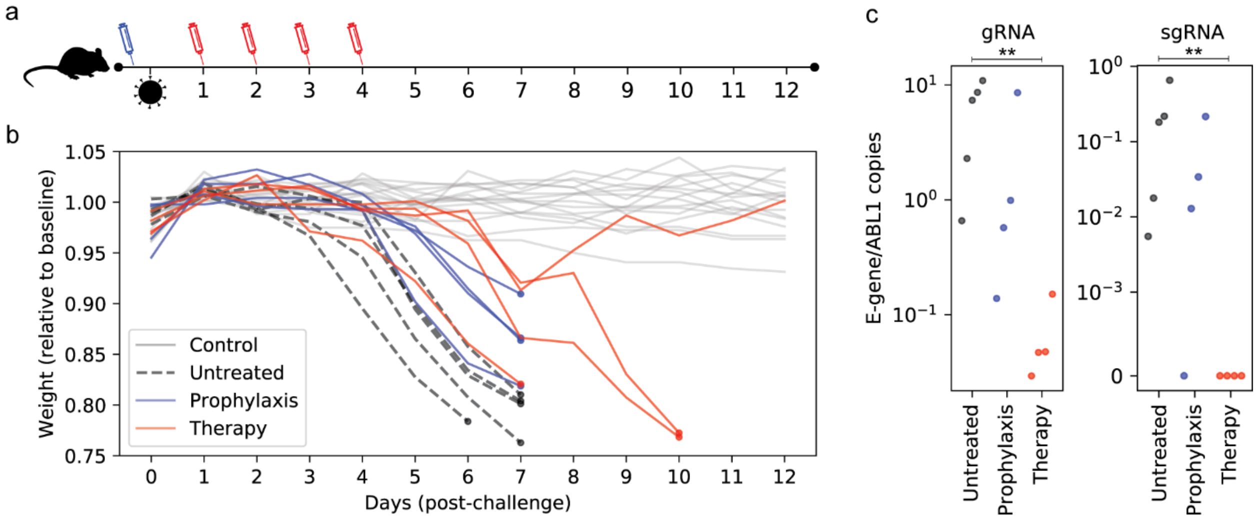

Fig. S9. Non-half-life extended nanobody heterodimer reduces disease severity in a SARS-CoV-2 challenge model.

(**a**) Timeline of the challenge experiment. K18-hACE2 transgenic mice were challenged with 1000 plaque forming units (PFU) of SARS-CoV-2 (Swedish isolate) and received prophylactic (blue) or therapeutic (red) Fu2-Ty1 at the indicated time points. (**b**) Weight of mice during the challenge experiment. The mean weight of each mouse of day 0 to day 2 served as baseline and the weight loss relative this baseline is shown. Uninfected mice are shown in grey, untreated infected mice in black, prophylactic treatment group in blue and therapeutic group in red. (**c**) Analysis of oropharyngeal samples from mice at day 6 in infected groups. Ratios of E-gene to ABL1 is shown for both genomic and subgenomic RNA. Infected mice treated with Fu2-Ty1 ($n = 4$ animals) harbored significantly reduced viral loads compared to untreated mice ($n = 5$ animals), evident for both genomic ($p = 0.0079$, Mann–Whitney $U = 0$, one-tailed) and subgenomic ($p = 0.0079$, Mann–Whitney $U = 0$, one-tailed) viral RNA.

Table S1. Cryo-EM data collection and processing

	**Dimer of spike trimer + 6 Fu2**	**Localized reconstruction of 2 RBDs + 2 Fu2**
**EMDB**	**EMD-12561**	**EMD-12465**
**PDB**	**7NS6**	**7NLL**
**Data collection**		
Microscope	TFS Krios G3i	
Voltage (kV)	300	
Detector	Bioquantum K3	
**Recording mode**	Counting	
EFTEM SA Magnification	165kx	
Calibrated pixel size (Å)	0.505	
Movie micrograph pixel size (Å)	0.505	
Flux (e^-/[(camera pixel)*s])	8.4	
Number of frames per movie micrographs	60	
Total movie micrograph exposure time (s)	1.5	
Fluency ($e^-/Å^2$)	48.6	
Underfocus range (μm)	0.2 – 1.3	
Energy filter slit width (eV)	10	
Data collection automation software	EPU	
**EM Data processing**		
Number of micrographs	14,081	14,081
Number of total projection images	1,035,962	1,035,962
Number of projection images used for reconstruction	277,372	277,372
Symmetry	C1	C1
Map resolution (FSC 0.143; Å)	3.18	2.9
Map resolution no mask (FSC 0.143; Å)	4.2	8.9
B-factor applied to map ($Å^2$)	68.1	37.3
**3D-FSC**[51]		
3D-FSC resolution (3D-FSC 0.143; Å)	4.0	3.2
Sphericity (3D-FSC)	0.831	0.924

Table S2. Cryo-EM model refinement and validation statistics

	Dimer of spike trimers + 6 Fu2	Localized reconstruction of 2 RBDs + 2 Fu2
**EMDB**	**EMD-12561**	**EMD-12465**
**PDB**	**7NS6**	**7NLL**
**Refinement**		
Refinement Package	PHENIX 1.19	PHENIX 1.19
Refinement space	Real	Real
Highest resolution used	3.2	2.9
**Model composition**		
Atoms refined	55904	5058
Residues SARS-CoV-2 Spike (or RBD) and nanobody Fu2	7109	656
Glycans	50	2
**Full Map-model correspondence**		
Map correlation coefficient	0.87	0.90
Map to model FSC (Å) (FSC 0.143)	3.2	2.6
**Mean B-factor**		
Residues SARS-CoV-2 Spike (or RBD) and nanobody Fu2	140	85.89
Glycans	129	96.22
**Model geometry**		
RMSD ideal bond length (Å)	0.004	0.003
RMSD ideal bond angles (˚)	0.826	0.529
Side chain rotamer outliers (%)	0	0
C-ß outliers (%)	0.38	0
**Ramachandran plot**		
Favored (%)	97.76	99.38
Outliers (%)	0	0
**General macromolecular model validation**		
MolProbity score[60]	1.66	1.38
Clashscore[60]	12.38	6.92
**EM specific model validation**		
CaBLAM (%)[61]	2.55	0.94
EM-ringer score[61]	1.73	3.16

Table S3. P values for data in Figure 4

Feature	Virus Challenge	Group A	Group B	Mann Whitney U	P (one-tailed)	
weight	WT	-	Fu2-Alb1	0	0,002	**
weight	Beta	-	Fu2-Alb1	0	0,003	**
weight	WT	Unchallenged	-	0	0,00000064	****
weight	WT	Unchallenged	Fu2-Alb1	9,5	0,0077	**
weight	Beta	Unchallenged	-	0	0,00000208	****
weight	Beta	Unchallenged	Fu2-Alb1	6,5	0,0034	**
VL sgE	WT	-	Fu2-Alb1	0	0,0143	*
VL sgE	Beta	-	Fu2-Alb1	2	0,1143	ns
VL E	WT	-	Fu2-Alb1	0	0,0143	*
VL E	Beta	-	Fu2-Alb1	2	0,1143	ns

Supplemental References

60. Chen, V. B. *et al.* MolProbity: All-atom structure validation for macromolecular crystallography. *Acta Crystallographica Section D: Biological Crystallography* **66**, 12–21 (2010).

61. Barad, B. A. *et al.* EMRinger: Side chain-directed model and map validation for 3D cryo-electron microscopy. *Nature Methods* **12**, 943–946 (2015).
