# Supplementary material for: A bispecific monomeric nanobody induces spike trimer dimers and neutralizes SARS-CoV-2 in vivo
Source: Nat Commun. 2022 Jan 10;13:155. doi: 10.1038/s41467-021-27610-z (PMC8748511; doi:10.1038/s41467-021-27610-z)
Supplement: Supplementary file 2 — Reporting Summary [file 41467_2021_27610_MOESM2_ESM.pdf]

nature portfolio

Corresponding author(s): B Martin Hällberg
Ben Murrell
Gerald McInerney

Last updated by author(s): Oct 18, 2021

Reporting Summary

Nature Portfolio wishes to improve the reproducibility of the work that we publish. This form provides structure for consistency and transparency in reporting. For further information on Nature Portfolio policies, see our Editorial Policies and the Editorial Policy Checklist.

Statistics

For all statistical analyses, confirm that the following items are present in the figure legend, table legend, main text, or Methods section.

n/a	Confirmed	
☐	☒	The exact sample size (*n*) for each experimental group/condition, given as a discrete number and unit of measurement
☐	☒	A statement on whether measurements were taken from distinct samples or whether the same sample was measured repeatedly
☐	☒	The statistical test(s) used AND whether they are one- or two-sided<br>*Only common tests should be described solely by name; describe more complex techniques in the Methods section.*
☒	☐	A description of all covariates tested
☒	☐	A description of any assumptions or corrections, such as tests of normality and adjustment for multiple comparisons
☐	☒	A full description of the statistical parameters including central tendency (e.g. means) or other basic estimates (e.g. regression coefficient) AND variation (e.g. standard deviation) or associated estimates of uncertainty (e.g. confidence intervals)
☐	☒	For null hypothesis testing, the test statistic (e.g. *F*, *t*, *r*) with confidence intervals, effect sizes, degrees of freedom and *P* value noted<br>*Give P values as exact values whenever suitable.*
☒	☐	For Bayesian analysis, information on the choice of priors and Markov chain Monte Carlo settings
☒	☐	For hierarchical and complex designs, identification of the appropriate level for tests and full reporting of outcomes
☒	☐	Estimates of effect sizes (e.g. Cohen's *d*, Pearson's *r*), indicating how they were calculated

Our web collection on statistics for biologists contains articles on many of the points above.

Software and code

Policy information about availability of computer code

Data collection	EPU (Thermo Fisher Scientific)
Data analysis	WARP, cryoSPARCv3.2.0, coot 0.9.6, phenix1.19.2, chimeraX-1.1.1, BIAevaluation, FoCuS-point, Prism 9

For manuscripts utilizing custom algorithms or software that are central to the research but not yet described in published literature, software must be made available to editors and reviewers. We strongly encourage code deposition in a community repository (e.g. GitHub). See the Nature Portfolio guidelines for submitting code & software for further information.

Data

Policy information about availability of data

All manuscripts must include a data availability statement. This statement should provide the following information, where applicable:

- Accession codes, unique identifiers, or web links for publicly available datasets
- A description of any restrictions on data availability
- For clinical datasets or third party data, please ensure that the statement adheres to our policy

The structure of the spike protein trimer PDB: 7KSG was used as a starting model for model building. The cryo-EM density maps have been deposited in the Electron Microscopy Data Bank under accession codes EMD-12561 (dimer of spike trimer + 6 Fu2) and EMD-12465 (localized reconstruction of 2 RBDs + 2 Fu2). The atomic coordinates have been deposited in the Protein Data Bank under IDs 7NS6 (dimers of spike trimers + 6 Fu2) and 7NLL (localized reconstruction of 2 RBDs + 2 Fu2). All nanobody sequences will be made available on public databases upon publication.

Field-specific reporting

Please select the one below that is the best fit for your research. If you are not sure, read the appropriate sections before making your selection.

[x] Life sciences [ ] Behavioural & social sciences [ ] Ecological, evolutionary & environmental sciences

For a reference copy of the document with all sections, see nature.com/documents/nr-reporting-summary-flat.pdf

Life sciences study design

All studies must disclose on these points even when the disclosure is negative.

Sample size	Previous experience with the K18-hACE2 challenge model suggested that weight trajectories (our primary readout) exhibit relatively low variance, and so sample sizes were selected to minimize the number of animals needed to attain statistical significance when treatment and control groups were relatively well separated. Explicit power calculations were not performed.
Data exclusions	No data were excluded from any analysis performed
Replication	Replicated experiments are indicated in the figure legends
Randomization	This study focuses on functional and structural characterization of a novel nanobody, and randomization is not relevant.
Blinding	This study focuses on functional and structural characterization of a novel nanobody, and blinding is not relevant.

Reporting for specific materials, systems and methods

We require information from authors about some types of materials, experimental systems and methods used in many studies. Here, indicate whether each material, system or method listed is relevant to your study. If you are not sure if a list item applies to your research, read the appropriate section before selecting a response.

Materials & experimental systems

n/a	Involved in the study	
[x]	[ ]	Antibodies
[ ]	[x]	Eukaryotic cell lines
[x]	[ ]	Palaeontology and archaeology
[ ]	[x]	Animals and other organisms
[x]	[ ]	Human research participants
[x]	[ ]	Clinical data
[x]	[ ]	Dual use research of concern

Methods

n/a	Involved in the study	
[x]	[ ]	ChIP-seq
[ ]	[x]	Flow cytometry
[x]	[ ]	MRI-based neuroimaging

Eukaryotic cell lines

Policy information about cell lines

Cell line source(s)	Cell lines were purchased from ATCC: HEK293T (ATCC-CRL-3216), Vero E6 (ATCC CRL-1586), Calu3 (HTB-55) or Thermo Fischer Scientific: FreeStyle 293-F
Authentication	Authentication was not performed, since all lines were recently purchased from ATCC
Mycoplasma contamination	All cell lines used for experiments were negative for Mycoplasma as determined by PCR.
Commonly misidentified lines (See ICLAC register)	No commonly misidentified cell lines were used in the study

Animals and other organisms

Policy information about studies involving animals; ARRIVE guidelines recommended for reporting animal research

Laboratory animals	New world camelid, 7 year old female alpaca (PreClinics, Germany). K18-hACE2 transgenic mice (Jackson Laboratories) were 11-26 weeks old at the start of the experiment (mean: 15.4 weeks). 13 males, 10 females.
Wild animals	This study did not involve wild animals
Field-collected samples	This study did not involve samples collected in the field.

Ethics oversight	All work involving alpaca immunizations at PreClinics GMBH complies with the relevant ethical regulations for animal testing and research. Ethical permits for studies of virus infection and therapeutic intervention were obtained from the Swedish Board of Agriculture (10513-2020)

Note that full information on the approval of the study protocol must also be provided in the manuscript.

Flow Cytometry

Plots

Confirm that:

- [x] The axis labels state the marker and fluorochrome used (e.g. CD4-FITC).
- [x] The axis scales are clearly visible. Include numbers along axes only for bottom left plot of group (a 'group' is an analysis of identical markers).
- [ ] All plots are contour plots with outliers or pseudocolor plots.
- [x] A numerical value for number of cells or percentage (with statistics) is provided.

Methodology

Sample preparation	HEK293T-hACE2 cells were trypsinized and fixed in 4% formaldehyde in PBS for 20 min. Cells were stained with spike-AbberiorStar-635P not premixed or premixed with Fu2 or control nanobody
Instrument	BD FACSCelesta
Software	FlowJo
Cell population abundance	No sorting was performed with the flow cytometer
Gating strategy	For gating forward scatter vs side scatter was used to separate cell from debris, resulting events were displayed as a histogram

- [x] Tick this box to confirm that a figure exemplifying the gating strategy is provided in the Supplementary Information.
